# Optimization of a hybrid bacterial/*Arabidopsis thaliana* fatty acid synthase system II in *Saccharomyces cerevisiae*

**DOI:** 10.1016/j.mec.2023.e00224

**Published:** 2023-06-15

**Authors:** Tatiana A. Pozdniakova, João P. Cruz, Paulo César Silva, Flávio Azevedo, Pier Parpot, Maria Rosario Domingues, Magnus Carlquist, Björn Johansson

**Affiliations:** a CBMA - Center of Molecular and Environmental Biology, University of Minho, Campus de Gualtar, Braga, 4710-057, Portugal; b CEB - C, University of Minho, Campus de Gualtar, 4710-057, Braga, Portugal; c LABBELS - Associate Laboratory, Braga, Portugal; e Centre of Chemistry, University of Minho, Campus de Gualtar, 4710-057, Braga, Portugal; f Mass Spectrometry Center & LAQV-REQUIMTE, Department of Chemistry, University of Aveiro, Campus Universitário de Santiago, 3810-193, Aveiro, Portugal; g CESAM–Centre for Environmental and Marine Studies, Aveiro, Portugal; h Department of Chemistry, University of Aveiro, Campus Universitário de Santiago, 3810-193, Aveiro, Portugal; i Division of Applied Microbiology, Lund University, Box 124, 221 00, Lund, Sweden

**Keywords:** Saccharomyces cerevisiae, E. coli, Fatty acid synthase, FASI, FASII, Metabolic engineering

## Abstract

Fatty acids are produced by eukaryotes like baker's yeast *Saccharomyces cerevisiae* mainly using a large multifunctional type I fatty acid synthase (FASI) where seven catalytic steps and a carrier domain are shared between one or two protein subunits. While this system may offer efficiency in catalysis, only a narrow range of fatty acids are produced. Prokaryotes, chloroplasts and mitochondria rely instead on a FAS type II (FASII) where each catalytic step is carried out by a monofunctional enzyme encoded by a separate gene. FASII is more flexible and capable of producing a wider range of fatty acid structures, such as the direct production of unsaturated fatty acids. An efficient FASII in the preferred industrial organism *S. cerevisiae* could provide a platform for developing sustainable production of specialized fatty acids. We functionally replaced either yeast FASI genes (*FAS1* or *FAS2*) with a FASII consisting of nine genes from *Escherichia coli* (*acpP*, *acpS* and *fab* -*A*, -*B*, -*D*, -*F*, -*G*, -*H*, -*Z*) as well as three from *Arabidopsis thaliana* (*MOD1*, *FATA1* and *FATB*). The genes were expressed from an autonomously replicating multicopy vector assembled using the Yeast Pathway Kit for *in-vivo* assembly in yeast. Two rounds of adaptation led to a strain with a maximum growth rate (μmax) of 0.19 h^−1^ without exogenous fatty acids, twice the growth rate previously reported for a comparable strain. Additional copies of the *MOD1* or *fabH* genes resulted in cultures with higher final cell densities and three times higher lipid content compared to the control.

## Highlights

• Thirteen fatty acid genes expressed simultaneously on a multicopy vector.
• Yeast fatty acid synthase replaced with genes from *E. coli* and *Arabidopsis thaliana*.
• Maximum growth rate (μmax) of 0.19 h–1.
• Additional gene copies of MOD1 (Enoyl-ACP reductase) or fabH improve growth.
• Three times more lipid accumulation than wild type.

## 1 Introduction

The concept of sustainable use of environmental resources originated from forestry in the 18th century, pertaining to how much wood may be extracted while still allowing equal yields at future harvests. Today, environmental concerns and fear of climate change highlight the necessity for transitioning from a fossil-carbon economy to a more sustainable circular bio-based economy (Lange et al., 2020). International political instability has recently severely impacted petroleum transportation and subsequent applications owing to the unequal dispersion and control of oil deposits across the earth. Alternatively, biosynthesis of molecules can be achieved from worldwide renewable resources using well-known microorganisms as cell factories (Mattanovich et al., 2014). Microbial production of fuels and fine chemicals is still under active development towards a feasible replacement of petroleum-based counterparts in a cost-efficient manner (Lawson et al., 2021; Nielsen and Keasling, 2016). In particular, fatty acids (FAs) and their activated forms, acyl-CoA or acyl-ACP, are precursors of a vast array of molecules with biotechnological and economical relevance that can vary in chain length and functional groups. FAs and FA-derived methyl/ethyl esters, hydroxy fatty acids, fatty alcohols, and fatty alkanes/alkenes can be used in the fabrication of detergents, cosmetics, plastics, fuels and other chemicals (Cho et al., 2020). FA biosynthesis is catalyzed by a Fatty Acid Synthase (FAS) system in an iterative process adding two carbons to the growing acyl chain in cycles of four reactions. The yeast *S. cerevisiae* expresses a type I FAS system characterized by a multifunctional enzyme composed of two distinct polypeptides that are coded by the genes *FAS1* and *FAS2*. Each of the polypeptides encloses several domains with specific roles in the mechanism (Schweizer and Hofmann, 2004). Contrarily, the FASII system, present in bacteria, organelles, algae, and protists, is composed of dispersed monofunctional polypeptides, each catalyzing a single reaction (Lu et al., 2004). Several type I FAS systems have been expressed in yeast resulting in altered fatty acid profiles (Table 1). FASI from *Mycobacterium vaccae*, in combination with a modified endogenous elongation system (Yu et al., 2017) or with short-chain-specific thioesterase (Zhu et al., 2020), produced very long-chain or medium-chain FAs, respectively (Table 1, #8 and #9). FA production in *S. cerevisiae* has also been attempted using FAS from *Homo sapiens* (Leber and Da Silva, 2014) (Table 1, #1), the FAS from several Actinomyces (Eriksen et al., 2015) (Table 1, #3), a hybrid fungal FAS (Zhu et al., 2017) (Table 1, #7), or a mutated FASI from yeast (Wernig et al., 2020) (Table 1, #10). In theory, FASII systems should allow greater flexibility of the resulting FAs by, for example, changing the thioesterase of the system, which controls the acyl chain length. The expression of type II FAS systems in *S. cerevisiae* have also been reported in four separate publications (Table 1, #2, #4, #5, #6). The separate expression of individual genes belonging to the FASII from *Streptococcus pyogenes* in yeast resulted in increased production of C16 and C18 fatty acids (Jung et al., 2015) (Table 1, #2). Another report describes the expression of the FASII from *E. coli* successfully providing lipoic acid (C8) for a heterologous pyruvate dehydrogenase (Lian and Zhao, 2016) (Table 1, #5). To our knowledge, only one study demonstrated the complementation of the native FAS system of *S. cerevisiae* through the expression of a FASII system (Fernandez-Moya et al., 2015) (Table 1, #4). A strain lacking the gene FAS2, and thus unable to synthesize FAs, grew without FAs supplementation when expressing the FASII from *E. coli*. The engineered strain produced a wider range of FAs, ranging from C14 and C18, while the wild-type strain produced nearly undetectable amounts of C14. These systems have provided evidence of their potential of both replacing the native FASI system and producing distinct end-products. The discrete nature of the FASII systems can confer greater control over each reaction and a means to synthesize FAs in a tailor-made fashion. The FASII yeast strain reported by Fernandez-Moya (Fernandez-Moya et al., 2015) and co-workers was based on the laboratory *S. cerevisiae* strain BY4741 (Brachmann et al., 1998), also widely used in the Saccharomyces Genome Deletion Project (Goffeau, 2000). The host strain had a deletion of *FAS2* while the *FAS1* gene was left intact. The expression of *FAS1* will occur independently of *FAS2* (Wenz et al., 2001), and contains acetyl-transacylase, dehydratase, enoyl-reductase, malonyl-transacylase, and palmitoyl-transacylase activities. It can not be ruled out that a heterologously expressed FASII interacts with one or more of the FAS1 domains, creating a functional hybrid. Further, the particular strain was created by at least five rounds of genetic integration separated by *lox*P-mediated marker recycling. Growth in medium without supplementation of fatty acids commenced after about 12 h of lag phase and was followed by relatively slow growth (μ = 0.093 h^−1^). There is a risk that slow growth could be caused by additional undesired genetic changes from multiple rounds of transformations and expression of the CRE recombinase (Solis-Escalante et al., 2015), something that is difficult to test as the heterologous genes are integrated into multiple locations in the genome. The aim of this work was to create a self-contained and (re-)movable genetic construct, able to express a FASII system in *S. cerevisiae* and to find if this construct can complement both *FAS1* or *FAS2* deletions. A secondary goal was to leverage the single yeast gene expression vectors created as a by-product of the Yeast Pathway Kit (Pereira et al., 2016) assembly to investigate the gene dosage effect in combination with strain adaptation as means to create a strain more amenable for further study.

**Table 1:** Published data on the expression of type I or type II Fatty Acid Synthase (FAS) systems in *Saccharomyces cerevisiae*; NA stands for “not applicable”.

#	Source	FAS type	Relevant FAs produced	Complements FAS^-^	Reference
1	*Homo sapiens*	I	Medium-chain FAs (C6-C12)	Yes	(Leber and Da Silva, 2014)
2	*Streptococcus pyogenes*	II	Long-chain FAs (C16-C18)	No	(Jung et al., 2015)
3	Actinomyces organisms	I	Long-chain fatty acids (C16-C18)	Yes	(Eriksen et al., 2015)
4	*Escherichia coli*	II	Long-chain FAs (C14-C18)	Yes	(Fernandez-Moya et al., 2015)
5	*Escherichia coli*	II	Octanoic acid (C8)	NA	(Lian and Zhao, 2016)
6	*S. cerevisiae* mitochondria	II	Failed expression	NA	(Lian and Zhao, 2016)
7	Hybrid fungal	I	Medium-chain FAs (C6-C12)	Yes	(Zhu et al., 2017)
8	*Mycobacterium vaccae*	I	Very long-chain FAs (C22-C26)	Yes	(Yu et al., 2017)
9	*Mycobacterium vaccae*	I	Medium-chain FAs (C6-C12)	Yes	(Zhu et al., 2020)
10	Mutated *S. cerevisiae* FASI	I	Octanoic acid (C8)	No	(Wernig et al., 2020)

## 2 Materials & methods

### 2.1 Microbial growth media

*Escherichia coli* strain XL1-Blue (Stratagene, La Jolla, CA, USA) was cultivated in Lysogeny-Broth-Miller medium “LB”; 10 g/L Tryptone (BD biosciences, San Jose, CA, USA), 5 g/L yeast extract and 10 g/L sodium chloride (Panreact AppliChem, Darmstadt, Germany) with 100 mg/L ampicillin (Formedium, King's Lynn, UK) when needed. *Saccharomyces cerevisiae* strains were cultivated in Yeast Extract–Peptone–Dextrose “YPD”; 10 g/L peptone (BD biosciences, San Jose, CA, USA), 10 g/L yeast extract (Panreact AppliChem, Darmstadt, Germany) and 20 g/L glucose (Scharlab S.L., Barcelona, Spain). *S. cerevisiae* was also cultivated in defined Synthetic Complete media “SC”; 1.7 g/L yeast nitrogen base w/o amino acids and w/o ammonium sulfate (BD, Franklin Lakes, NJ, USA), 5.0 g/L ammonium sulfate (Panreact AppliChem, Darmstadt, Germany), 20 g/L glucose (Scharlab S.L., Barcelona, Spain) and 1.4 g/L Kaiser amino acid drop-out mixture (Formedium, King's Lynn, UK) without histidine, leucine, tryptophan and uracil (Kaiser et al., 1994). SC media was supplemented with 80 mg/L L-histidine, 400 mg/L L-leucine, 80 mg/L L-tryptophan and 80 mg/L uracil. Leucine (SC-U) or leucine and uracil (SC-L-U) were omitted as required for selection for auxotrophic markers. Geneticin (G418 Sulfate; Gibco™, Scotland, UK) was added to a concentration of 0.2 g/L for selection of *FAS1* or *FAS2* gene deletion mutants. *S. cerevisiae* strains with non-functional fatty acid synthase were cultivated with 460 mg/L of myristic acid (HMy; C14; ≥99% Sigma-Aldrich®, Saint Louis, MO, USA) and 10 g/L of polyoxyethylene sorbitan monopalmitate (Tween 40, ACROS Organics™, Thermo Fisher Scientific, Belgium). For solid medium, 20 g/L of agar (LabChem Inc, Zelienople, PA, USA) was added.

### 2.2 Construction of the pTA1 vector

The pTA1 vector was made by homologous recombination from five PCR products obtained from four different vectors, each providing functional characteristics. The pBR *E. coli* origin of replication and the *amp*R ampicillin resistance gene were amplified from pBR322 (Bolivar et al., 1977). The pBR origin of replication retained the ROP gene in order to limit copy number in *E. coli*. A deletion allele of the *E. coli crp* gene (CRPΔ) was amplified from pYPKpw (Pereira et al., 2016). The *S. cerevisiae* gene *LEU2* was amplified from YEplac181 (Gietz and Sugino, 1988). Finally, the yeast 2μ origin of replication was amplified from YEplac112 (Gietz and Sugino, 1988). Each PCR primer carried 30 bp long tails enabling homologous recombination between the fragments. The Primer tails were computationally designed orthogonal spacer sequences designed using the R2oDNA tool (Casini et al., 2014).

### 2.3 Construction of the pYPK0_FASII expression vector

The Yeast Pathway Kit (Pereira et al., 2016) protocol was used to assemble and express FASII pathways in *S. cerevisiae*. The FASII pathway consisted of a linear assembly of thirteen tandemly expressed genes, each controlled by a unique promoter. Gene sequences from fatty acid synthase of type II (FASII) were amplified from *E. coli* XL1-Blue genomic DNA and from *A. thaliana* Col-0 cDNA. PCR products corresponding to individual gene open reading frames were cloned into the blunt *Aji*I site in the *E. coli* suicide vector pYPKa or into the same site in the *E. coli/S. cerevisiae* shuttle vector pYPK0 by *in-vivo* gap repair directed by primer tails to guide homologous recombination. *S. cerevisiae* intergenic sequences from tandemly expressed genes were amplified from pYPKa vectors harboring these sequences cloned in either of two blunt restriction sites, *Zra*I or *EcoR*V. Promoter, gene and terminator sequences were amplified from vectors by three pairs of PCR primers specific for the plasmid backbone so that PCR products share short stretches of flanking homology. Thirteen different single gene expression vectors were made by homologous recombination between a specific promoter, gene and terminator. The single gene expression vectors were made so that the terminator in one was the same intergenic fragment as the promoter for another, for all but the first and the last cassette in the assembly. This design choice facilitates the recombination of all cassettes into a compact pathway directed by the shared promoters and terminators. The expression cassette from each single gene expression vector was PCR amplified and recombined with the pYPKpw vector linearized with restriction endonuclease *Zra*I creating the pYPK0_FASII (*URA3*) or pTA1 linearized with restriction endonuclease *FspA*I creating pTA1_FASII (*LEU2*). Both vectors express all thirteen genes simultaneously (Table 2). The cloning procedure has been expressed in Jupyter notebooks (Kluyver et al., 2016) using the general programming language Python with pydna (Pereira et al., 2015). These notebooks as well as the DNA sequences of final and intermediate constructs are available in a version-controlled git repository hosted by github (bit.ly/Pozdniakova_2023).

**Table 2:** Promoters, genes and terminators used to express genes in pYPK0_FASII or pTA1 with genes expressed in previously published work for comparison.

#	Promoter	Gene	Terminator
1	PDC1	EcfabH	TEF1
2	TEF1	EcfabD	FBA1
3	FBA1	EcfabG	RPL22A
4	RPL22A	EcacpP	TDH3
5	TDH3	EcfabF	UTR2
6	UTR2	EcfabB	TPI1
7	TPI1	EcfabA	PMP3
8	PMP3	EcfabZ	ENO2
9	ENO2	AthMOD1	RPL5
10	RPL5	AthFATA1	RPL16A
11	RPL16A	AthFATB	RPL17A
12	RPL17A	EcacpH	RPL16B
13	RPL16B	EcacpS	TMA19

### 2.4 Construction of the pTA1_FASIIb expression vectors

The pTA1_FASIIb (24,902 bp) vector is similar to the pTA1_FASII but for the omission of the *E. coli acpH* gene. The vector was made by homologous recombination between eight PCR products ranging in size from 911 bp to 3934 bp derived from and covering almost the entire pTA1_FASII vector. Divergent primers on each side of the *acp*H gene facilitated homologous recombination excluding the gene.

### 2.5 Strain construction

The *S. cerevisiae* strain CEN. PK2-1C (*MATa ura3_52 his3_Δ1 leu2_3112 trp1_289, MAL2_8c SUC2*) was used as the starting point for strain construction (Table 3). The *FAS1*/YKL182W beta and FAS2/YPL231W alpha subunits of fatty acid synthetase were deleted in separate strains by the introduction of a KanMX4 cassette (Güldener et al., 1996) targeted for either loci. These strains were designated CENΔfas1 and CENΔfas2 respectively. Yeast strains were transformed with plasmids as indicated in Table 3.

**Table 3:** *Saccharomyces cerevisiae* strains used in this work.

Strain	Relevant information
CEN.PK2-1C	MATa ura3-52 his3-Δ1 leu2-3,112 trp1-289 MAL2-8c SUC2 (Entian and Kötter, 1998)
CEN.PK2-1C.pTA1	CEN.PK2-1C transformed with plasmid pTA1 (empty vector) Leu+
CEN.PK2-1C.FASIIb	CEN.PK2-1C transformed with plasmid pTA1_FASIIb Leu+
CENΔfas1	CEN.PK2-1C fas1:KanMX4 G418R
CENΔfas2	CEN.PK2-1C fas2:KanMX4 G418R
CENΔfas1.pTA1	CENΔfas1 transformed with plasmid pTA1 (empty vector) Leu + G418R
CENΔfas2.pTA1	CENΔfas2 transformed with plasmid pTA1 (empty vector) Leu + G418R
CENΔfas1.FASIIb	CENΔfas1 transformed with plasmid pTA1_FASIIb Leu + G418R
CENΔfas2.FASIIb	CENΔfas2 transformed with plasmid pTA1_FASIIb Leu + G418R

### 2.6 Strain adaptation to medium without fatty acids

CENΔfas2. FASIIb was transformed with twelve different single gene expression (TU) vectors, selecting for uracil prototrophy, resulting in twelve strains carrying two plasmids which were stored at −80 °C in 50% glycerol. The twelve strains, CENΔfas1. FASIIb and CENΔfas2. FASIIb were plated on solid YPD medium from frozen cultures and incubated at 30 °C for 1–3 days. A 50 mL FALCON tube with 5 mL YPD medium supplemented with G418 was inoculated and incubated at 30 °C and 200 rpm in an orbital shaker for six days. A small volume (300 μL) of cells was removed and used to inoculate 5 mL of fresh medium of the same type. The initial optical density of the culture was measured and when four duplications were reached, 100 μL of the culture was transferred to a new tube with 5 mL of the same medium. The last culture transfer was repeated six times. A small amount of the final culture was spread on solid SC-L-U medium. One clone of each strain was collected and stored at −80 °C in 50% glycerol.

### 2.7 Aerobic batch culture

Strains CENΔfas1. FASIIb and CENΔfas2. FASIIb were used to inoculate YPD medium supplemented with G418 or SC-L followed by incubation at 30 °C. Cells of these cultures were used to inoculate fresh medium followed by incubation at 30 °C. Cells were allowed to divide at least three times, reaching at least eight times the initial OD600. Cells were subsequently transferred to a fresh culture. This procedure was repeated three times. Clones isolated from each culture had greatly reduced lag time for both strains and were used for the physiological analysis of substrate consumption and product formation (Fig. 3). Liquid SC medium without leucine (SC-L) was inoculated with *S. cerevisiae* CEN. PK2-1C.pTA1 (empty vector), CEN. PK2-1C.pTA1_FASIIb, CENΔfas1. FASIIb or CENΔfas2. FASIIb. Cultures were incubated for 72 h (30 °C and 200 rpm) and harvested by centrifugation (4500×*g* for 4 min). Subsequently, 20 mL cultures in 100 mL Erlenmeyer flasks were inoculated to a uniform OD_600_ of 0.5, corresponding to 1.5 x10⁷ cells/mL. Each strain was cultivated in twenty four identical Erlenmeyer flasks. Three flasks were incubated for measuring cell density throughout the experiment (165h). Every 24h, three flasks for each strain were removed for measurements of OD_600_ and pH, lipid estimation by fluorimetry and flow cytometry, carbon source consumption and by-product formation by high-performance liquid chromatography (HPLC) analysis. Intracellular lipids were quantified by gravimetry and fatty acids were analyzed by gas chromatography measured for cells collected at the last time point.

### 2.8 Analytical methods

Biomass concentration in submerged cultures was measured as optical density at 600 nm (OD_600_) using the spectrophotometer Genesys 20 (Thermo Fisher Scientific Inc) and pH was measured using the Crison Instruments™ pH electrode 5202 and pH meter Crison Instruments™ micro pH 2002. Intracellular lipids were estimated by a fluorometric assay using Fluoroskan Ascent® FL (Thermo Fisher Scientific Inc) after staining with lipophilic dye Nile red (>98.0% Sigma Aldrich, Steinheim, Germany) (Miranda et al., 2020). The Nile red fluorophore is excited at 515 nm and emits at 585 nm. A volume of 200 μL of Nile red stained cells was collected by centrifugation (15,700 RCF; 30s; Eppendorf 5415D) and washed with 200 μL PBS. Cells were fixed by resuspending in 100 μL of PBS 1x: Formaldehyde (0.96:0.04 v/v) for 15 min to 1 h. Cells were then collected, washed with 1 mL PBS 1x, resuspended in 200 μL PBS 1x and stored at 4 ^O^C until analysis. Fixed cells were analyzed by flow cytometry analysis was performed using a Beckman Coulter Cytoflex flow cytometer with a PE-A detector, 200 μL of stained cells suspensions were pipetted to 96 well black flat bottom polystyrene microplates (Costar®, Corning, ME, USA), as for fluorimetry, cells suspension without Nile red stain and cells suspension with known lipid content were used as controls. Glucose consumed and fermentation byproducts formed were determined by high-performance liquid chromatography with refractive index detection (HPLC-RI). The samples were prepared as described previously (Bettencourt et al., 2020) and separated with a HyperREZ™ XP Carbohydrate H+ 8 μm (Thermo Electron Corporation, Waltham, MA, USA) column on a LaChrom Elite® (VWR Hitachi, Tokyo, Japan) chromatography system with a LaChrom Elite® L-2490 RI detector (VWR Hitachi, Tokyo, Japan). The mobile phase was 2.5 mM of sulfuric acid at a flow rate of 0.5 mL/min and the column was held at 45 °C. The sulfuric acid solution was diluted from concentrated H_2_SO_4_ (95–97%, Merck KGaA, Darmstadt, Germany) in ultrapure water and filtered by a vacuum pump through 0.22 μm acetate cellulose membrane prior to use. Ultrapure water was produced by Milli-Q® system and deionized water by Elix® system (Merck KGaA, Darmstadt, Germany). The lipid content by dry cell weight (DCW; % w/w) was determined by gravimetry according to (Miranda et al., 2020). The lipids extracted were then subjected to an acid-catalyzed transesterification process as described by (Bettencourt et al., 2020) and the lipid fraction was determined by gas chromatography with a flame ionization detector (GC-FID; CP-3800 Varian, Agilent, Santa Clara, CA, USA) using a 30 m × 0.25 mm × 0.25 μm Teknokroma® TR WAX column, with helium as carrier gas at 1 mL/min. The injector and detector temperatures were 250 °C while for the column the first 2 min was at 50 °C followed by a heating ramp of 10 °C/min up to 225 °C that was then maintained for 10 min. The volume of the injected sample was 1 μL with a split of 1:20 and the results were expressed as percentages of total FAs detected. For peak identification, a mixture containing 37 fatty acid methyl esters ranging from C4:0 to C24:1 was used (Supelco™ 37 Component FAME Mix, Sigma Aldrich, Steinheim, Germany). All data were expressed as the mean of three replicates ± standard deviations.

## 3 Results

### 3.1 Native FASI system disruption and complementation

**Fig. 1:**
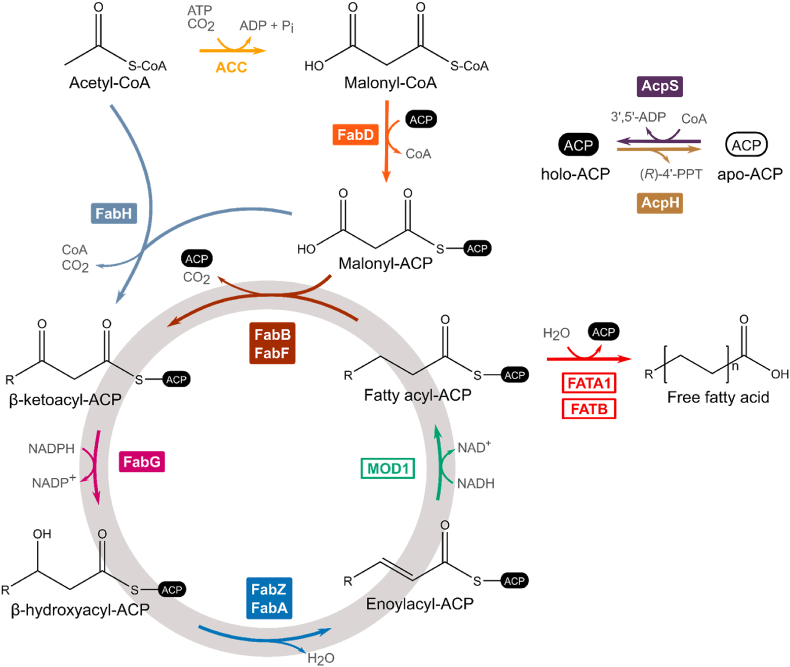
Schematic representation of the hybrid FASII system expressed in *Saccharomyces cerevisiae*. The illustration depicts ten enzymes of the FASII pathway, two enzymes for the Acyl Carrier Protein (ACP) apo/holo conversion, the ACP coded by the gene *acpP*, and the Acetyl-Coa Carboxylase from yeast (ACC). Solid gene labels represent enzymes from *Escherichia coli* and open labels represent enzymes from *Arabidopsis thaliana*.

The *FAS1*/YKL182W or *FAS2*/YPL231W genes were deleted from *S. cerevisiae* CEN. PK2-1C by integrating a *Kan*MX4 cassette specific for either locus, resulting in two FAS-deficient strains designated CENΔfas1 and CENΔfas2, respectively. Both strains required supplementation with FAs for growth (results not shown). Thirteen genes (*fabH*, *fabD*, *fabG*, *acpP*, *fabF*, *fabB*, *fabA*, *fabZ*, *acpS* and *acpH* from *Escherichia coli* and, *MOD1*, *FATA1* and *FATB* from *Arabidopsis thaliana*) were PCR amplified from *E. coli* XL1-Blue (Stratagene) chromosomal DNA or *A. thaliana* Columbia-0 cDNA (Fig. 1). The *MOD1* gene was chosen instead of the corresponding *E. coli* gene (*fabI*) since *MOD1* has a higher codon adaptation index for *S. cerevisiae* and seven rare codons compared to 23 for the *fabI* gene and was thus considered to have a higher chance of efficient expression (Fig. S1). The *FATA1* and *FATB* encode acyl-acyl carrier protein (ACP) thioesterases largely specific for oleoyl-ACP (C18) and palmitoyl-ACP (C16) (Salas and Ohlrogge, 2002). These are the two main fatty acids found in *S. cerevisiae* so production of these would maximize the chances of successful complementation of the yeast native FASI. Furthermore, plant acyl-ACP thioesterases resulted in high free fatty acid production in *E. coli* (Zhang et al., 2011) and was also efficient in the context of FASII expression in *S. cerevisiae* (Fernandez-Moya et al., 2015). The FASII genes were cloned in single gene transcriptional unit vectors by yeast *in-vivo* homologous recombination each with a unique promoter and terminator, following the Yeast Pathway Kit strategy (Pereira et al., 2016). The sequence of each transcriptional unit was confirmed by diagnostic PCR and partial DNA sequencing. The promoters and terminators were intergenic sequences from tandemly expressed *S. cerevisiae* genes. All thirteen transcriptional units were joined by homologous recombination between promoters and terminators and the *S. cerevisiae/E. coli* shuttle vector pYPKpw resulting in plasmid pYPK0_FASII (25,707 bp), expressing thirteen genes simultaneously. The vector showed signs of genetic instability when maintained in *E. coli* for preparation of pure plasmid DNA (results not shown). This instability could be due to the large size of the plasmid which might be pushing the upper limit of stable maintenance in *E. coli.* The pYPKpw vector (Pereira et al., 2016) is maintained in *E. coli* by the pUC origin of replication, lacking the ROP gene of the pBR322 and with a point mutation further increasing copy number. The pTA1 vector was constructed with the sequences needed for compatibility with the Yeast Pathway Kit but with a *LEU2* marker for selection in *S. cerevisiae* and a complete pBR origin of replication including an intact ROP gene. The same pathway was assembled in a similar manner, but with the pTA1 vector instead of the pYPKpw resulting in the pTA1_FASII vector. The pTA1_FASII (26,279 bp) vector, although slightly larger, was stable enough to allow purification and diagnostic restriction digestion in order to confirm the structure of the pathway.

**Fig. 2:**
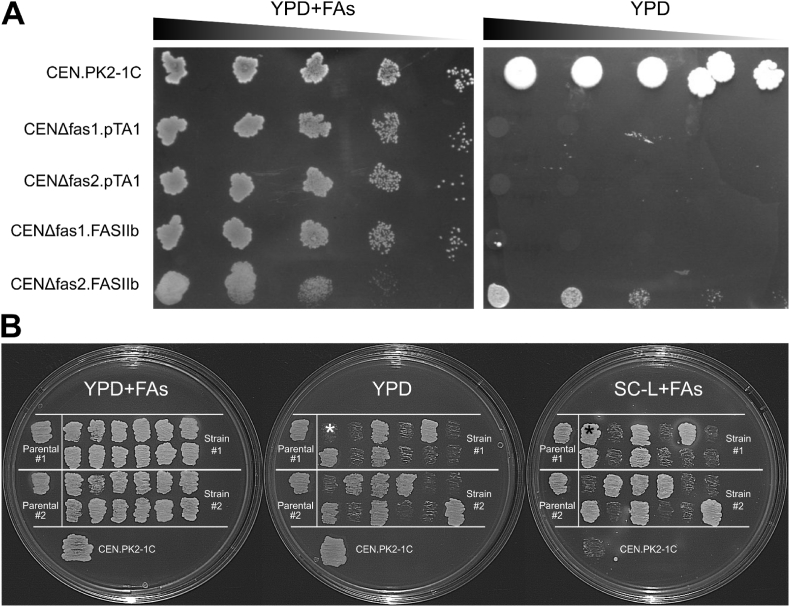
Growth on media with and without FA supplementation. **A**. 10-fold serial dilutions of *S. cerevisiae* (CEN.PK2-1C) and FASI mutants transformed with an empty plasmid (CENΔfas1. pTA1 and CENΔfas2. pTA1) or with the pTA1_FASIIb (CENΔfas1. FASIIb and CENΔfas2. FASIIb) were plated on solid YPD media with FAs or without FAs. The diffuse appearance of the droplets on the left pane is due to the oily texture of the medium. **B.** Growth on different media after three passages on non-selective rich media supplemented with FA. An isolate that lost the ability to grow without supplemented FAs but retained leucine prototrophy is indicated with *.

The CENΔfas1 and CENΔfas2 strains were transformed with pTA1_FASII or pTA1 (empty vector) to provide suitable negative controls in the complementation assay. Both strains grew on medium supplemented with FAs but neither on medium without FA supplementation (Fig. 2A). The pTA1_FASII pathway contains the entire *E. coli* ACP processing machinery including the structural ACP gene, *acpP*, the holo-ACP synthase, *acpS*, and the ACP phosphodiesterase, *acpH* (Fig. 1). The synthase transfers the (R)-4′-phosphopantetheine ((R)-4′-PPT) moiety of CoA to the apo-ACP to form holo-ACP, the active form of ACP, while the phosphodiesterase catalyzes the opposite reaction (Thomas et al., 2007; Thomas and Cronan, 2005). We hypothesized that *acpH* could be detrimental to the FASII due to misregulation in *S. cerevisiae*. The inhibition could be specific for FASII by not allowing enough active ACP for FASII to function or there could be a toxic effect of apo-ACP, observed earlier in *E. coli* (Keating et al., 1995). The pTA1_FASII plasmid was reconstructed without the *acpH* gene resulting in a vector called pTA1_FASIIb (24,902 bp), expressing all other genes from pTA1_FASII. The CENΔfas1 and CENΔfas2 strains were transformed with pTA1_FASIIb resulting in strains CENΔfas1. FASIIb and CENΔfas2. FASIIb. These strains were able to grow on solid YPD (Fig. 2A) or SC media without FA supplementation (results not shown), indicating that the pTA1_FASIIb successfully complemented the inactive *FAS1* or *FAS2* genes. A lower proportion of CENΔfas1. FASIIb cells grew under selective conditions compared to complementation of CENΔfas2. FASIIb (Fig. 2A). Three individual colonies of the CENΔfas1. FASIIb were observed in the highest concentration droplet. This shows that the pTA1_FASIIb pathway can complement a fas^−^ phenotype whether caused by a deletion of *FAS1* or *FAS2*. The pTA1_FASIIb was lost from CENΔfas2. FASIIb by three sequential transfers on YPD medium supplemented with FA. Fourteen isolates out of twenty four lost the ability to grow without FA supplementation concomitantly with loss of growth without leucine (Fig. 2B), indicating that the phenotype depends solely on the pTA1_FASIIb plasmid. Plasmid pTA1 carries the *LEU2* markers and reverts leucine auxotrophy in *leu*2 strains. Only one isolate grew without leucine with auxotrophy for FA, possibly due to rearrangements in the plasmid (Fig. 2B, marked with an asterisk). FA prototrophy combined with leucine auxotrophy was not observed.

### 3.2 Batch cultivation of strains expressing FASII

**Fig. 3:**
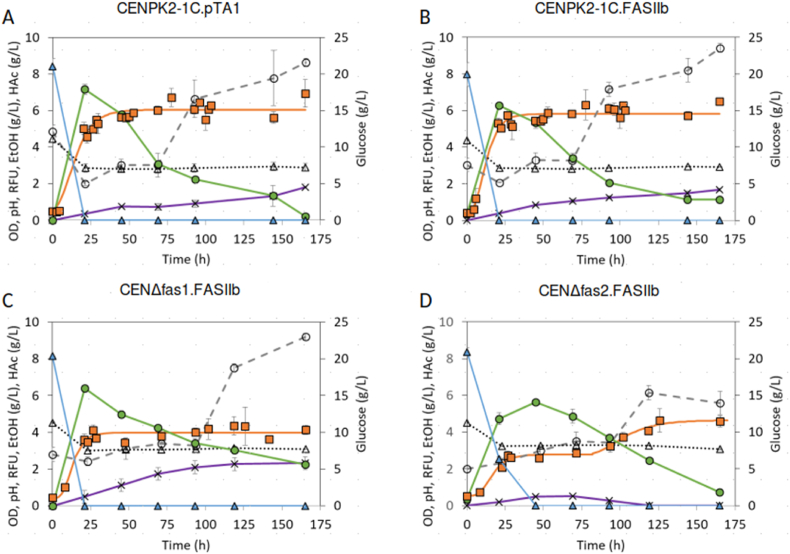
Substrate consumption, product formation and intracellular lipid accumulation in aerobic batch cultures. Results are expressed as mean concentration (g/L) ± standard deviation of triplicates. Symbols: □֊ OD, ··Δ·· pH, ֊Δ֊ glucose, -ο- EtOH (green), -x- HAc, -ο- RFUs (Relative Fluorescent Units). (For interpretation of the references to colour in this figure legend, the reader is referred to the Web version of this article.)

Four yeast strains, CENPK2-1C.pTA1, CENPK2-1C.FASIIb, CENΔfas1. FASIIb and CENΔfas2. FASIIb (Table 2), were grown to stationary phase in liquid SC-L medium and then used to inoculate 20 mL of the same medium to an initial OD_**600**_ = 0.5 in a 100 mL Erlenmeyer flask (Fig. 3). The two first strains retained an active native FASI transformed with the empty pTA1 vector or the pTA1_FASIIb pathway vector. Samples were withdrawn and analyzed periodically between 0 and 165 h with measurements of OD_**600**_, pH, glucose, ethanol and acetate concentrations and relative fluorescent units (RFU) with Nile red. Growth commenced after 4–5 h for the CENPK2-1C.pTA1 and CENPK2-1C.FASIIb while the CENΔfas1. FASIIb and CENΔfas2. FASIIb took 6–8h. The CENΔfas1. FASIIb and CENΔfas2. FASIIb grew to a final OD_**600**_ of about 4 while the CEN strains retaining the native FASI grew to about 6 (Fig. 3A and B). Interestingly, stationary phase was reached after about 25 h for all strains except CENΔfas2. FASIIb which went through a diauxic growth with an interesting slowdown between 50 and 75 h (Fig. 3D). Low amounts (1–2 g/L) of acetic acid were produced by all strains. The end of the exponential phase coincided with glucose exhaustion and peak ethanol concentration but for the CENΔfas2. FASIIb (Fig. 3D) where the second growth phase coincides with acetic acid exhaustion. Overall, the presence of the FASIIb pathway in any strain seemed to lower the ethanol titer somewhat from 7.2 g/L (Fig. 3A) to around 6 g/L (Fig. 3B, C and D) where theoretical yield would give 10.2 g/L from 20 g/L glucose. A pH decrease from 4.4 to 2.9 was observed for all strains during the exponential phase. Curiously, there was a duplication in the RFU reading as ethanol concentration drops below about 3.7 g/L in all cases. Four times as much glycerol accumulated in strains with the FASII pathways compared to the CEN. pTA1 strain. The maximum glycerol concentration for the CENPK2-1C.pTA1 reached 0.50 ± 0.05 g/L while the strains with the FASIIb pathway reached over 2 g/L. Overall, the observations are compatible with a redox imbalance caused by the FASIIb pathway as glycerol secretion is often a sign of NADH surplus (Geertman et al., 2006). CENΔfas1. FASIIb and CENΔfas2. FASIIb produced small amounts of propionic acid (<0.6 g/L, results not shown).

#### 3.2.1 Fatty acid characterization

Intracellular lipids were extracted after 165 h incubation (last time point in Fig. 3), quantified and characterized (Table 4). Lipids were extracted at a high yield as can be inferred from the extraction using commercial olive oil as a control (91%). Strains with an inactive native FASI system (CENΔfas1. FASIIb and CENΔfas2. FASIIb) produced slightly more unsaturated and less saturated LCFAs than strains retaining the FASI (CENPK2-1C.pTA1 and CENPK2-1C.FASIIb). While all strains synthesized about 15% of saturated and about 84% of unsaturated LCFAs, the ratio for strains with an inactive native FASI system was 0.19 while strains with a native FASI system showed a ratio of 0.17. Curiously, there is little discernible difference between an empty plasmid and the FASIIb pathway when the FASI system is intact. The native FASI system may be better suited to the intracellular environment with more efficient processing of substrates, masking the presence of the FASIIb pathway. The main difference between any of the strains with native FASI and the two strains relying on FASIIb was the amount of palmitoleic (C16:1n7) and oleic (18:1n9c) acids produced. The former produced around 50% palmitoleic acid and around 30% oleic acid, while the latter produced around 75% palmitoleic and only around 4% oleic acid along with small amounts of saturated and monounsaturated C14 fatty acids. These results partly contrast with results in a comparable system where a FASII pathway operates as the only fatty synthase (Fernandez-Moya et al., 2015). In the previous study, the same decrease in C18 fatty acids was observed, but C14 fatty acids made up a larger portion of the total fatty acids at 10–20%. Incidentally, the FASII pathway used in the study had little effect on fatty acid synthesis when paired with a functional FASI pathway in concordance with our observations. Two *A. thaliana* Acyl-CoA thioesterases were expressed in the FASIIb pathway, FATA1 and FATB. The former has been reported to hydrolyze mainly C16-ACP while the latter hydrolyses C18-ACP. The increase of C16 fatty acids observed could result from a balance between FATA1 and FATB in the FASIIb context or it could reflect an inherent limitation in the chain length extending capacity of the FASIIb pathway. Small amounts of odd-chain pentadecanoic C15 fatty acids were observed in strains relying on the FASIIb pathway. Small amounts of propionic acid have been detected in *S. cerevisiae* fermentation (Eglinton et al., 2002). *S. cerevisiae* acetyl-CoA synthetase (ACS) can catalyze the formation of propionyl-CoA from propionate (Jones and Lipmann, 1955; Pronk et al., 1994). The *E. coli* FabH, responsible for the transacylation of acetyl-CoA to Acetyl-ACP in the FASIIb pathway can also use propionyl-CoA albeit with lower efficiency (Choi et al., 2000; Heath and Rock, 1996) providing a possible route for the formation of odd chain LCFA.

**Table 4:** Long-chain fatty acid profiles (% of total FAs) obtained from yeasts after 165h of growth at 30 °C and 200 rpm (Fig. 3). The results are the mean ± SD of triplicates. FAs with concentrations of more than 1% are shown.

	C14:0	C14:1	C15:0	C16:0	C16:1	C18:0	C18:1n9	C18:2n6	Sum (%)
	Myristic acid	Myristoleic acid	Pentadecanoic acid	Palmitic acid	Palmitoleic acid	Stearic acid	Oleic acid	Linoleic acid	
CEN.PK2-1C				9.6 ± 0.3	50.7 ± 0.3	4.6 ± 0.2	34.1 ± 0.6		99
CEN.pTA1				9.7 ± 0.6	52 ± 3	4.4 ± 0.4	32 ± 2		98
CEN.FASIIb				10.3 ± 0.2	49.5 ± 0.1	4.82 ± 0.05	34.0 ± 0.4		99
CENΔfas1.FASIIb	1.20 ± 0.05		1.07 ± 0.7	12.7 ± 0.3	74.1 ± 0.6	1.4 ± 0.3	8.2 ± 0.3		98
CENΔfas2.FASIIb	2.08 ± 0.05	1.14 ± 0.03	1.02 ± 0.04	11 ± 1	72 ± 2	3 ± 1	9.0 ± 0.4		99
Olive oil				16.9 ± 0.1	1.23 ± 0.06	2.5 ± 0.01	71.2 ± 0.1	7.05 ± 0.03	99

### 3.3 Metabolic optimization of FASIIb system by gene dosage

The FASIIb pathway expresses FASII genes from a selection of *S. cerevisiae* promoters so there is not likely any mechanism by which the cell can regulate FASIIb genes on the transcriptional level. We, therefore, designed an experiment to probe gene dosage effects for each of the genes in the pathway. The TU vectors containing a single gene expression cassette that was a by-product of the assembly of the pYPK0_FASII pathway vector were reintroduced resulting in twelve strains (Table S1) each carrying a vector with an extra copy of a gene already present in the pTA1_FASIIb pathway. The resulting strains were cultivated in selective medium (SC-L-U) for seven rounds of growth and adaptation as described before. As each strain took different amounts of time to reach the last culture, each strain was stored at −80 °C until all strains completed the growth regiment. The strains were streaked on solid medium and used to inoculate YPD medium to an identical initial optical density. The adapted strains were cultivated for three days in YPD medium after which strains carrying extra copies of *MOD1* or *fabH* were found to have grown to the same optical densities as the CEN. PK2-1C control strain (7 ± 2), while the adapted CENΔfas2. FASIIb reached about half this density (3.9 ± 0.2).

**Fig. 4:**
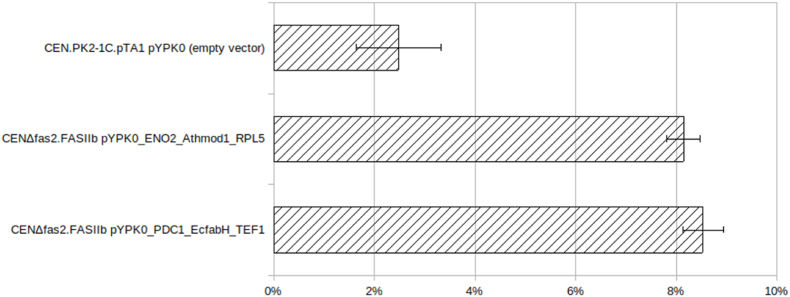
Total lipid content by % dry weight for three strains that underwent seven rounds of adaptation to media without added FA.

**Fig. 5:**
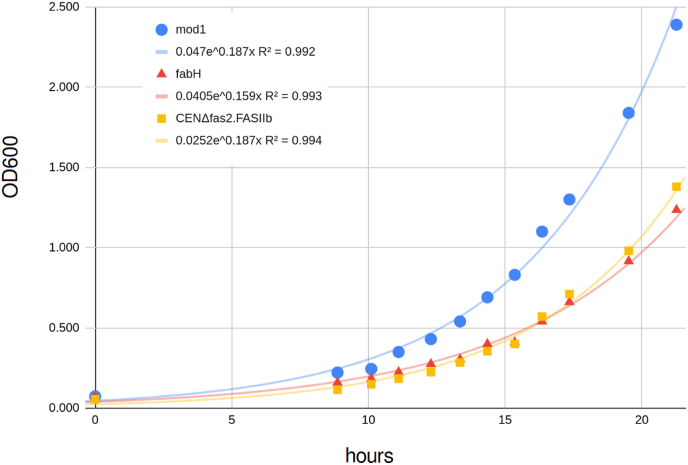
Measurement of growth rate in aerobic batch cultures of CENΔfas2. FASIIb with and without extra copies of *MOD1* or *fabH*. An exponential growth model (OD_600_(t) = OD_600_ (t = 0) * e^t^) was fitted to the data (solid lines).

The CENΔfas2. FASIIb strain with MOD1 or FabH were found to have about three times the intracellular lipid content (Fig. 4) compared to the CEN. PK2-1C.pTA1 with an empty pYPK0 vector. Initial aerobic growth rates in YPD media were measured for the adapted CENΔfas2. FASIIb with extra copies of *MOD1* or *fabH* (Fig. 5). The CENΔfas2. FASIIb with or without *MOD1* vector grew at 0.187 h^−1^ while the CENΔfas2. FASIIb with the *fabH* vector grew at 0.159 h^−1^. These growth rates compare favorably to rates reported before (Fernandez-Moya et al., 2015) where the growth model used here returns a rate of 0.093 h^−1^. It should be noted that the genes, while largely the same (Table 5) were integrated, possibly providing lower gene dosage than the multicopy vectors used in the present study.

**Table 5:**
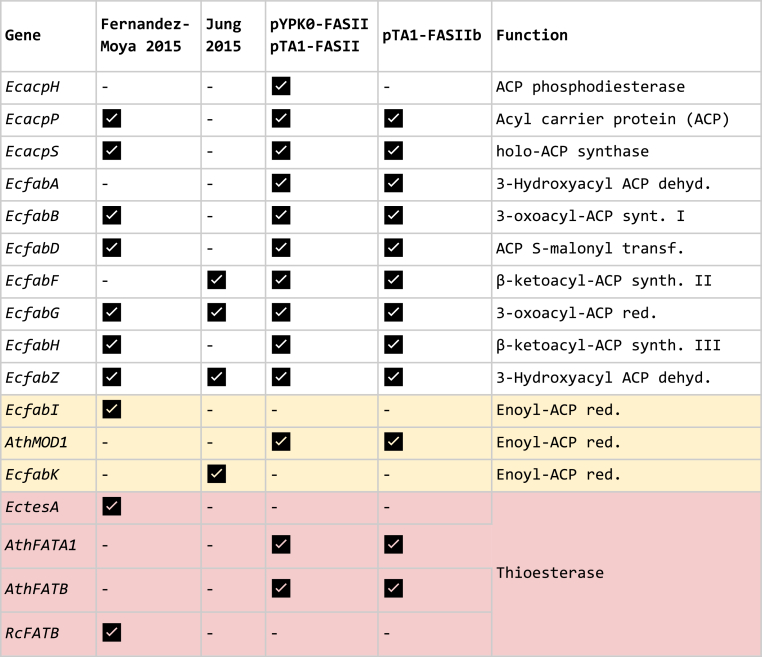
Genes expressed in pYPK0_FASII or pTA1_FASII with genes expressed in previously published work for comparison.

## 4 Discussion

Deletion of either of the type I FAS structural genes (*FAS1* and *FAS2*) lead to a complete dependency on the pTA1_FASIIb pathway for growth without exogenously added FAs. We did observe that the complementation was seemingly more efficient for the *FAS2* deletion than the *FAS1* as far fewer clones appeared on medium w/o FAs for the *FAS1* deletion strain. As *FAS1* induces the expression of *FAS2* (Wenz et al., 2001), there may be very little *FAS2* gene product in a *fas1* deletion strain while there might be *FAS1* gene products in a *fas2* deletion strain. Our results do not rule out an interaction between the *FAS1* encoded α-subunit and the FASIIb pathway. However, we also observed that CENΔfas1. FASIIb grew as well as CENΔfas2. FASIIb after adaptation, indicating that there is a way for the strain to adapt to the imposed limitation. The pTA1_FASIIb expresses two additional genes, *fabA* and *fabB* compared to a previously published FASII expression construct in yeast (Table 5). In contrast to FabZ, FabA has both dehydratase and isomerase activity and is involved in the synthesis of unsaturated fatty acids by isomerizing to a *cis* 3-alkene which is not processed by the subsequent enoyl reductase. While FabB and FabF have similar specificities for elongation, FabB seems necessary for the elongation of *cis*-3-decenoyl-ACP (C10) (Cronan et al., 1969) which is the precursor for C16:1 and C18:1 unsaturated fatty acids in *E. coli*. We observed a shift toward C16:1 monounsaturated fatty acids at the expense of C18:1 while the total amount ratio of saturated vs monounsaturated FAs remained about the same. A previously published comparable strain (Fernandez-Moya et al., 2015) produced considerably less C18:1 fatty acids, consistent with a role for the FabA and FabB in FASIIb similar to the one in *E. coli.* An efficient FAS system II fatty acid synthase in *S. cerevisiae* could potentially be a means for producing specialized fatty acids not efficiently produced by the native FASI system. We observed that expression of the native FASI and FASIIb simultaneously seem to negate the effect of the FASIIb pathway as the fatty acid pattern produced is very similar to the one of the host strain. A previously published FASII expression construct in yeast (Fernandez-Moya et al., 2015) showed a similar result. This effect may be associated with expression of FASI and FASII is the same compartment, something that is unique to these strains. Further experiments with downregulation of the native FASI might clarify this issue. Another challenge would be to increase the total amount of fatty acids being produced. Many strategies have been implemented to increase the level of FAs production in *S. cerevisiae*. Upregulating pathways that lead to higher availability of the precursors acetyl-CoA (Lian et al., 2014; Tang et al., 2013) and malonyl-CoA (Pereira et al., 2022; Runguphan and Keasling, 2014), and of cytosolic NADPH (de Jong et al., 2014), downregulating competing pathways (Ghosh et al., 2016), or preventing lipid degradation (Leber et al., 2015; Zhou et al., 2016) have proven to be efficient approaches.

## 5 Conclusions

In this work, the successful complementation of *Saccharomyces cerevisiae* FAS1 or FAS2 was demonstrated using a FAS system II pathway carried on a yeast episomal vector and that this vector was necessary and sufficient for the reversion of the non-growth phenotype. The selection on media without exogenous fatty acids produced strains that grow at about half the rate of the concomitant strain with an intact FAS1 and 2, which may serve as a development tool for more efficient FASII pathways in yeast. The pathway can also be used to explore other yeast backgrounds such as robust industrial yeast strains to explore the natural range of metabolic capacity in this respect.

## Author statement

All persons who meet authorship criteria are listed as authors, and all authors certify that they have participated sufficiently in the work to take public responsibility for the content, including participation in the concept, design, analysis, writing, or revision of the manuscript.

Furthermore, each author certifies that this material or similar material has not been and will not be submitted to or published in any other publication.

## Declaration of competing interest

The authors declare that they have no known competing financial interests or personal relationships that could have appeared to influence the work reported in this paper.

## Data Availability

Data will be made available on request.
