# Supplementary material for: Optimization of a hybrid bacterial/Arabidopsis thaliana fatty acid synthase system II in Saccharomyces cerevisiae
Source: Metab Eng Commun. 2023 Jun 15;17:e00224. doi: 10.1016/j.mec.2023.e00224 (PMC10320613; doi:10.1016/j.mec.2023.e00224)
Supplement: Multimedia component 1 [file mmc1.docx]

| **Gene** | **cds** | **len** | **cai** | **gc** | **sta** | **stp** | **n-end** | **CGA** | **CGG** | **CGC** | **CCG** | **CTC** | **GCG** | **rare** |
| --- | --- | --- | --- | --- | --- | --- | --- | --- | --- | --- | --- | --- | --- | --- |
| FabI | ATG...TAA | 263.0 | 0.629 | 0.532 | 1.0 | 0.47 | >30 h | 0 | 0 | 8 | 6 | 2 | 7 | 0.087 |
| Mod1 | ATG...TAG | 318.0 | 0.694 | 0.455 | 1.0 | 0.23 | >30 h | 3 | 1 | 0 | 1 | 1 | 1 | 0.022 |

Figure S1.

Coding sequence for *E. coli* FabI (789 bp, CP123963 REGION: 2104605..2105393) and *A. thaliana* Mod1 (954 bp, NM_126612 REGION: 419..1370) analyzed for properties potentially important for succesful expression in *S. cerevisiae.* Codon adaptaion index (**cai**) was calculated using the pydna python package (<https://github.com/BjornFJohansson/pydna>). The codons CGA, CGG, CGC, CCG, CTC and GCG are relatively rare in *S. cerevisiae.* The code to generate the figure can be found in the Github repository below (*).

(*) <https://github.com/MetabolicEngineeringGroupCBMA/Pozdniakova_et_al_2022>

| *base strain* | TU plasmid | Extra gene |
| --- | --- | --- |
| CENfas2Δ.FASIIb | pYPK0_ENO2_AthMOD1_RPL5 | *mod1* |
| “ | pYPK0_PDC1_EcfabH_TEF1 | *fabH* |
| “ | pYPK0_UTR2_EcfabB_TPI1 | *fabB* |
| “ | pYPK0_TEF1_EcfabD_FBA1 | *fabD* |
| “ | pYPK0_TDH3_EcfabF_UTR2 | *fabF* |
| “ | pYPK0_FBA1_EcfabG_RPL22A | *fabG* |
| “ | pYPK0_RPL22A_EcacpP_TDH3 | *acpP* |
| “ | pYPK0_RPL16A_AthFATB_RPL17A | *fatB* |
| “ | pYPK0_RPL5_AthfatA1_RPL16A | *fatA1* |
| “ | pYPK0_RPL16B_EcacpS_TMA19 | *acpS* |
| “ | pYPK0_PMP3_EcfabZ_ENO2 | *fabZ* |
| “ | pYPK0_TPI1_EcfabA_PMP3 | *fabA* |

Table S1.

Strains carrying two plasmids, the pTA1_FASIIb (LEU2) vector containing the FASII metabolic pathway and a pYPK0 derivative with a single expression cassette (URA3).
