# Supplementary material for: Optimization of a hybrid bacterial/Arabidopsis thaliana fatty acid synthase system II in Saccharomyces cerevisiae
Source: Metab Eng Commun. 2023 Jun 15;17:e00224. doi: 10.1016/j.mec.2023.e00224 (PMC10320613; doi:10.1016/j.mec.2023.e00224)
Supplement: Multimedia component 2 [file mmc2.zip › mec_224_pTA1_FASIIb_mmc2.html]

./.html
